# Role of Toll-Like Receptors and Their Downstream Molecules in the Development of Nonalcoholic Fatty Liver Disease

**DOI:** 10.1155/2010/362847

**Published:** 2011-01-17

**Authors:** Kouichi Miura, Ekihiro Seki, Hirohide Ohnishi, David A. Brenner

**Affiliations:** ^1^Department of Gastroenterology and Neurology, Akita University Graduate School of Medicine, 1-1-1 Hondo Akita-shi, Akita 010-8543, Japan; ^2^Department of Medicine, School of Medicine, University of California, San Diego, 9500 Gilman Drive, La Jolla, CA 92093, USA

## Abstract

Activation of innate immunity is associated with the development of liver disease, including non-alcoholic fatty liver disease (NAFLD). In the innate immune system, Toll-like receptors (TLRs) are sensors that recognize bacterial and viral components such as lipopolysaccharide, bacterial DNA, and peptidoglycan. Recent data have demonstrated that the liver is exposed to a high load of TLR ligands due to bacterial overgrowth and increased intestinal permeability in NAFLD. Upon stimulation by these TLR ligands, hepatic immune cells produce various mediators that are involved in host defense. On the other hand, these mediators alter lipid metabolism, insulin signaling, and cell survival. Indeed, some TLR-deficient mice demonstrate lesser degrees of NAFLD even though TLR ligands are increased. This paper will highlight the recent progress on the study of TLR signaling and their downstream molecules in the development of NAFLD.

## 1. Introduction

Nonalcoholic steatosis is a component of metabolic syndrome, and obese people with insulin resistance frequently have fatty liver disease [1]. Although steatosis is considered a benign liver disease, a subset of steatosis includes a progressive liver disease, nonalcohol steatohepatitis (NASH), that causes liver cirrhosis and cancer. In 1980, Ludwig et al. proposed the concept of “NASH”, steatohepatitis without a history of excess alcohol intake [2]. Currently, the term “nonalcoholic fatty liver disease (NAFLD)” is more widely used because it is difficult to diagnose NASH at an early stage without histological examinations. Thus, NAFLD comprises a spectrum of disorders ranging from simple steatosis to advanced steatohepatitis and fibrosis. Since NAFLD has become the most common liver disease and the prevalence is estimated to be 14–24% of the population in developed countries [3–5], NAFLD is a growing public health concern worldwide. 

 Hepatic steatosis occurs when the amount of imported and synthesized lipids exceeds the export or catabolism in hepatocytes [6–8], including (1) increased lipid delivery to the liver, (2) increased lipid uptake in hepatocytes, (3) increased de novo lipogenesis in the liver, (4) failure of lipid export, and (5) impaired hepatic mitochondrial *β*-oxidation of fatty acids. Steatosis is present in most patients with insulin resistance, suggesting that dysfunction of insulin signaling is closely associated with excessive accumulation of lipid in the liver. However, hepatic inflammation and consequent fibrosis are not always observed in these patients, suggesting that additional factors are required for the development of NASH. 

 This paper highlights Toll-like receptors (TLRs) and their downstream targets, including inflammatory cytokines and chemokines, as emerging factors in the development of NAFLD. We further review the role of nuclear factor *κ*B (NF-*κ*B) and c-Jun N-terminal kinase (JNK), key molecules mediating TLR signaling in NAFLD. Since hepatic resident macrophages, Kupffer cells, perceive various TLR ligands and produce inflammatory mediators through NF-*κ*B and/or JNK activation [9–11], we will focus on TLR signaling in Kupffer cells.

## 2. The Gut-Liver Axis Is an Important Pathway in the Development of NAFLD

 Gut microbiota, consisting of 15,000–35,000 species of bacteria, play a crucial role in nutrient absorption and energy storage [12–14]. Young conventionally reared mice have a 40% higher body fat and 47% higher gonadal fat content than germ-free mice, even though conventionally reared mice consume fewer calories. In addition, gnotobiotic mice exhibit a 60% increase in body fat within 2 weeks following transplantation of the gut microbiota from conventionally reared mice [15], indicating that gut microbiota contribute to nutrient acquisition. In particular, gut microbiota promote absorption of monosaccharides from the gut lumen, with resulting induction of de novo hepatic lipogenesis [15]. 

 In addition to nutrient acquisition, gut microbiota are a source of bacterial products such as lipopolysaccharide (LPS), bacterial DNA, and peptidoglycan, which are delivered to the liver through the portal vein. In murine models of NAFLD, bacterial overgrowth is observed with compositional change as well as increased intestinal permeability by reducing the expression of tight junction proteins such as ZO-1 and occludin [16]. In human, the composition of gut microbiota differs between individuals with and without diabetes mellitus [17, 18]. Indeed, circulating bacterial components are elevated in NAFLD patients and in animal models [19–22]. As a result, liver cells are exposed to a high load of bacterial products that function as TLR ligands. Since TLR signaling is a key pathway to produce inflammatory cytokines and chemokines, the gut microbiota contribute to the development of NAFLD as a source of TLR ligands.

## 3. TLRs Are Associated with NAFLD

 TLRs are associated with liver diseases including alcoholic liver injury, ischemia/reperfusion liver injury, liver fibrosis, and liver cancer [23, 24]. Among 13 TLRs identified in mammals, TLR2, TLR4, and TLR9 play a role in the development of NAFLD [20, 21, 25, 26]. To date, no information is available on the role of other TLRs in NAFLD. Results from gene-modified mice indicate that TLR4, and TLR9 signaling promote the progression of NAFLD. 

 Several groups have demonstrated that TLR4 signaling worsens NAFLD [19–21]. TLR4 is the receptor for LPS, a component of the Gram-negative bacterial cell wall. Serum LPS levels are increased in patients with hepatic steatosis caused by total parenteral nutrition or intestinal bypass [27–29]. Antibiotics treatment in these patients attenuates steatosis with decreased plasma levels of LPS [27–29]. Circulating LPS levels are elevated in most animal models of NAFLD induced by diets, including the high-fat (HF) diet, fructose-rich diet, methionine/choline-deficient (MCD) diet, and choline-deficient amino acid-defined (CDAA) diet [19–22]. Wild-type (WT) mice fed these diets show severe steatosis or steatohepatitis. In contrast, TLR4 mutant mice on these diets have less steatosis or steatohepatitis, although LPS levels are equivalent to those in WT mice. Even in mice on standard laboratory chow, continuous subcutaneous infusion of low-dose LPS results in hepatic steatosis, hepatic insulin resistance, and hepatic weight gain [30]. In addition, an intraperitoneal injection of LPS exacerbates liver injury in mice fed an MCD diet [31]. Eighty percent of intravenously injected LPSs molecules are detected in the liver within 20–30 min [32, 33]. These data indicate that the liver is the main target of LPS, and LPS-TLR4 is a key pathway for the progression of NAFLD.

 TLR9 signaling contributes to the development of NASH [26]. TLR9 recognizes DNA containing an unmethylated-CpG motif that is highly expressed in bacteria-derived DNA [34]. Although bacterial DNA is detectable in blood and ascites in patients with advanced cirrhosis [35, 36], it remains unclear whether bacterial DNA is present at the early stage of liver disease and whether bacterial DNA contributes to NAFLD. We have recently demonstrated that bacterial DNA is detectable in the blood in a murine model of NASH, and that bacterial DNA binding to TLR9 contributes to the development of steatohepatitis [26]. WT mice on a CDAA diet showed severe steatohepatitis with insulin resistance. In contrast, TLR9-deficient mice had less steatohepatitis even though bacterial DNA was present in the blood [26]. In addition, TLR9-deficient mice demonstrated less insulin resistance and less fibrogenic response [26]. 

 The role of TLR2 in NAFLD has not been well studied. TLR2 recognizes components of Gram-positive bacterial cell wall such as peptidoglycan and lipoteichoic acid [34]. At present, no studies have shown increased TLR2 ligands in NAFLD, which might be limited by current methodology. Blockade of TLR2 signaling prevents insulin resistance in HF diet-fed mice [37, 38]. In contrast, TLR2-deficient mice on an MCD diet exhibit equivalent levels of steatohepatitis but more severe steatohepatitis after LPS challenge compared to WT mice [25]. 

 MyD88 is a key molecule in the development of metabolic syndrome including NAFLD [39, 40]. MyD88, an adaptor protein for all TLRs except for TLR3, is required for the expression of various inflammatory cytokines and chemokines [41]. MyD88-deficient mice are protected from metabolic syndrome including atherosclerosis [39, 40] and from liver injury induced by bile duct ligation or carbon tetrachloride [23, 42]. We have demonstrated that MyD88-deficient mice on a CDAA diet show less steatohepatitis with less insulin resistance compared with WT mice [26]. As expected, inflammatory cytokines and fibrogenic factors are also significantly suppressed in MyD88-deficient mice compared with WT mice fed a CDAA diet [26].

## 4. Endogenous TLR Ligands in NAFLD

 Nonbacterial substances may function as TLR ligands; free fatty acids (FFAs) and denatured host DNA activate TLR2, TLR4 and TLR9 [43–46]. For instance, palmitate activates WT macrophage but not TLR4-deficient macrophages [44]. Stearic acid and palmitic acid, potential TLR4 ligands, are rich in dietary fat, and circulating FFAs are elevated in patients with NAFLD [47]. These data demonstrate an association between TLR4 and FFAs. On the other hand, some reports have demonstrated that FFAs do not bind to TLR4 [48, 49]. LPS has a high affinity for lipids such as chylomicrons and fatty acids, suggesting that contaminated LPS in the lipids may be the actual TLR4 ligand. Although the LPS-lipids complexes still have affinity to TLR4, the toxic effect of LPS is decreased [50, 51]. Thus, the concept of lipids as endogenous TLR4 ligands is still unresolved. TLR4 also recognizes oxidized phospholipid [52] and HMGB-1 [53]. To date, the role of these TLR4 ligands has not been investigated in NAFLD.

 Denatured host DNA is a candidate for a TLR9 ligand in liver injury. Apoptotic hepatocyte DNA induces type I collagen and TGF*β* expression in hepatic stellate cells via TLR9 [45]. Denatured host DNA also stimulates sinusoidal endothelial cells to produce interleukin (IL)-1*β* via TLR9 [24]. In these studies, TLR9-deficient mice were resistant to carbon tetrachloride- or acetaminophen-induced sterile liver injury. If apoptotic host DNA functions as a TLR9 ligand, NASH livers are constantly exposed to TLR9 ligands because hepatocytes undergo apoptosis and necrosis in NASH. However, the unmethylated CpG-motif is uncommon in mammalian DNAs [54], and host DNA is recognized by other DNA sensors such as DNA-dependent activator of IFN-regulatory factors and the inflammasome which sense cytosolic DNA in TLR9-indendent manner [55, 56]. Although some FFAs and denatured host DNA are attractive candidates for TLR ligands, further investigations are necessary to determine whether these nonbacterial substances function as reliable TLR ligands in NAFLD.

## 5. Liver Cells That Perceive TLR Ligands

**Figure 1:**
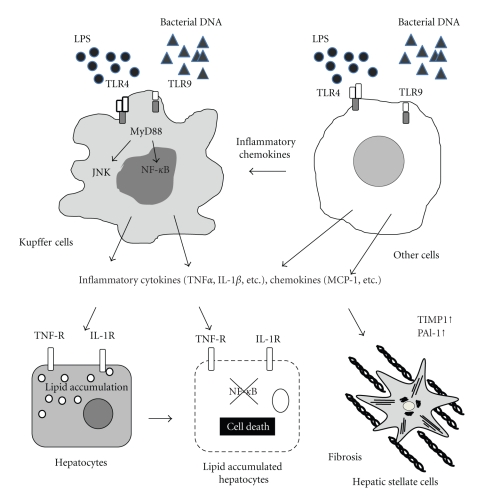
TLRs and downstream signaling in NAFLD. Kupffer cells respond to TLR ligands such as LPS and bacterial DNA through TLR4 and TLR9, respectively. Upon TLR ligation, MyD88, an adaptor molecule, is recruited to transmit the signals that activate NF-*κ*B and JNK. Activated Kupffer cells produce inflammatory cytokines such as TNF*α* and IL-1*β* and chemokines such as MCP-1 (CCL2). These inflammatory cytokines and chemokines induce lipid accumulation in hepatocytes and cell death. In addition, TNF*α* and IL-1*β* promote liver fibrosis by activating hepatic stellate cells. Other cells including hepatic resident cells, infiltrated cells into the liver, and adipose tissue macrophages produce various mediators in response to TLR ligands. These pathways also contribute to the development of NAFLD.

 The liver is composed of various types of cells including hepatocytes, biliary epithelial cells, hepatic stellate cells, Kupffer cells, and sinusoidal endothelial cells. Most types of liver cells are reported to express TLRs and produce various inflammatory mediators in response to TLR ligands [10]. For instance, hepatic stellate cells and sinusoidal endothelial cells produce chemokines and inflammatory cytokines in response to a TLR4 ligand [23] and a TLR9 ligand [24], respectively. Among resident liver cells, Kupffer cells are well documented to respond to various TLR ligands such as peptidoglycan, double-stranded RNA, LPS, bacterial DNA, and probably other TLR ligands. In addition, Kupffer cells are a major source of inflammatory cytokines such as TNF*α* and IL-1*β* [9, 11]. These cytokines produced by Kupffer cells promote lipid accumulation and cell death in hepatocytes as described below in detail. These cytokines also induce hepatic stellate cells to produce profibrogenic factors such as TIMP1 and PAI-1 [26, 57, 58]. Thus, Kupffer cell-derived mediators through TLRs affect lipid metabolism, liver damage and liver fibrosis in NAFLD (Figure 1). Indeed, depletion of Kupffer cells ameliorates the progression of diet-induced steatohepatitis. Rivera et al. have reported Kupffer cell depletion delayed the development of steatohepatitis induced by an MCD diet [20]. We also have shown that depletion of Kupffer cells decreased inflammatory cytokines in mice on a CDAA diet, resulting in improvement of NASH [26]. These findings indicate that Kupffer cells play a pivotal role in the development of steatohepatitis. On the other hand, the roles of Kupffer cells in HF diet models, a simple steatosis model, are more complicated. While most of reports have shown that depletion of Kupffer cells ameliorates steatosis [59–62], one report shows an opposite effect [63]. This discrepancy may partially depend on the methodology to deplete Kupffer cells. Clodronate liposome was used to deplete Kupffer cells by intravenous injection [61] or intraperitoneal injection [62, 63]. Intravenous injection selectively depletes Kupffer cells and/or splenic macrophages but not visceral fat macrophages whereas intraperitoneal injection affects both Kupffer cells and visceral fat macrophages [23, 64]. Adipose tissue macrophages are activated in an HF diet model [63] and release various mediators such as TNF*α* and IL-6, which influence insulin signaling and lipid metabolism. These mediators could further activate Kupffer cells, and contribute to steatosis. Although further studies are necessary to determine the role of adipose tissue macrophages in the development of NAFLD, it is clear that Kupffer cells are important in the development of NAFLD.

## 6. NF-*κ*B Activation in NAFLD

 Activation of the transcriptional factor NF-*κ*B, a downstream target for TLR-MyD88 signaling, is crucial for the inflammatory response in immune cells and is a key in the development of NAFLD [10, 11]. In NAFLD patients as well as animal models of NAFLD, NF-*κ*B activation is observed in liver cells, including hepatocytes, hepatic stellate cells and Kupffer cells [23]. Hepatocytes respond minimally to TLR ligands in vivo, suggesting that other mediators activate NF-*κ*B in hepatocytes [23, 65]. For instance, TNF*α* and IL-1*β* activate NF-*κ*B in hepatocytes [26, 66]. On the other hand, TLR ligands directly activate NF-*κ*B in Kupffer cells. TLR signaling triggers inflammatory cytokine and chemokine production in Kupffer cells through NF-*κ*B activation [26, 67]. IKK*β* activates NF-*κ*B by the phosphorylation and subsequent degradation of I*κ*-B, an essential inhibitor for NF-*κ*B. Specific deletion of IKK*β* in myeloid cells including macrophages results in suppression of inflammatory cytokine production, which prevents systemic insulin resistance induced by an HF diet [68]. 

 It is unclear whether NF-*κ*B activation in hepatocytes leads to steatosis. Hepatocyte-specific IKK*β* overexpression induces steatosis [69]. In contrast, NF-*κ*B essential modulator (NEMO) deficiency in hepatocytes results in spontaneous steatohepatitis [70]. NEMO deficiency completely blocks NF-*κ*B activation, indicating that NF-*κ*B activation in hepatocytes is not a primary cause of steatosis. We and others have recently demonstrated that hepatocytes increase their lipid content in response to TNF*α* and IL-1*β* [26, 71]. In that process, TNF*α* and IL-1*β* activate NF-*κ*B in normal hepatocytes. On the other hand, NF-*κ*B activation by TNF*α* and IL-1*β* is blunted in lipid-laden hepatocytes [26]. NF-*κ*B activation in hepatocytes may be required only for an initial step of lipid accumulation in the liver. Regardless of NF-*κ*B activation in hepatocytes, inflammatory cell infiltrations and expression of F4/80, a marker for macrophage, are increased in both hepatocyte-specific IKK*β* overexpressed mice and hepatocyte-specific NEMO-deficient mice [69, 70]. These data further support the concept that NF-*κ*B activation in immune cells is a key event in the development of NAFLD.

## 7. JNK Activation in NAFLD

 TLR-MyD88 signaling pathway activates JNK, a member of mitogen-activated protein kinases. JNK is an attractive target in the pathogenesis of NAFLD, because JNK activation plays a central role in the development of obesity and insulin resistance [72]. In patients and animals with NASH, JNK is activated in the liver, and JNK activation in immune cells results in inflammatory cytokine production [22]. Current research is analyzing the distinct roles of the JNK isoforms, JNK1 and JNK2, in the development of the metabolic syndrome including NAFLD. JNK1 promotes steatosis and inflammation in two different models of NAFLD [73]. In contrast, lack of JNK2 promotes liver injury [74]. However, it must be noted that the roles of JNKs are different in hepatocytes and Kupffer cells; JNK activation in hepatocytes is involved in cell death and insulin signaling whereas JNK activation in Kupffer cells induces inflammatory cytokine production. Recently, we and others have demonstrated the role of hematopoietic cells in the development of the metabolic syndrome including NASH [22, 75]. The results from chimeric mice generated by transplanting bone marrow cells lacking JNK1 or JNK2 into WT mice have shown that JNK1 in hematopoietic cells contributes to developing metabolic syndrome by producing inflammatory cytokines. Thus, hematopoietic cells including Kupffer cells and recruited macrophages play a pivotal role in the development of NAFLD. On the other hand, JNK in hepatocytes is involved in cell death and insulin signaling. Thus, JNK plays multiple roles in multiple steps in NAFLD.

## 8. Inflammatory Cytokines and NAFLD

 Inflammatory cytokines are important mediators in the development of NAFLD. Among inflammatory cytokines, TNF*α* and IL-1*β* have multiple functions including immune modulation, cell differentiation, proliferation, apoptosis, and energy metabolism. Indeed, expressions of TNF*α* and IL-1*β* are increased in NAFLD patients and animal models [76–79]. In contrast, most of TLR-deficient mice show decreased TNF*α* and IL-1*β* levels in NAFLD models [20, 26]. 

 TNF*α* levels are increased in the liver, the adipose tissue, and the serum of NAFLD patients [76, 77]. Expression of TNF receptors is also increased in the liver of NAFLD patients [77]. Mice deficient in both TNF receptor type 1 and type 2 demonstrate less steatosis, inflammation, and liver fibrosis in a NASH diet model [80], indicating that TNF receptor signaling contributes to the development of NAFLD. So far, several mechanisms of TNF*α*-mediated functions are proposed: (1) insulin resistance, (2) release of fatty acids from adipose tissue, (3) regulation of lipid influx and efflux in hepatocytes, and (4) hepatocyte cell death. TNF*α* impairs insulin signaling by suppressing insulin receptors, insulin receptor substrate-1 and GLUT4 expressions [80], and by the expression of SOCS-3. As a result of insulin resistance, FFAs and glucose uptake are inhibited in adipocytes, whereas increased insulin levels promote FFA flux into hepatocytes and hepatic lipogenesis [81]. Moreover, TNF*α* increases fatty acid release from adipose tissue by promoting lipolysis, resulting in insulin resistance. In addition to impaired insulin signaling and FFA metabolism, TNF*α* promotes cholesterol accumulation in hepatocytes by inducing expression of LDL receptor and by inhibiting efflux of cholesterol [71]. Thus, TNF*α* promotes lipid accumulation in hepatocytes inducing insulin resistance, increased FFA levels, and lipid retention in the cells. Lipid-accumulated hepatocytes are vulnerable to various stimuli such as TNF*α*. In NASH patients, hepatocyte apoptosis and necrosis frequently occur. TNF*α* stimulation alone does not induce cell death in normal hepatocytes, because TNF*α* induces the upregulation of NF-*κ*B-related antiapoptotic genes [66]. However, impaired lipid metabolism leads to hepatocyte apoptosis in the presence of TNF*α*. Hepatocytes laden with lipids have increased susceptibility to TNF*α*-induced cell death [82, 83]. Free cholesterol accumulation in hepatocytes depletes mitochondrial glutathione. This induces ROS generation in hepatocytes and then evokes cell death signaling [82]. In addition, lipid-accumulated hepatocytes increase the expression of ASK-1 and JNK in response to TNF*α* [83], which lead to cell death. These findings demonstrate that TNF*α* plays an important role in lipid metabolism as well as hepatocyte cell death in the development of NAFLD. 

 Increased IL-1*β* is recognized as a risk factor for the metabolic syndrome [84]. Indeed, expression of IL-1*β* as well as its receptor is increased in the adipose tissue of obese patients with type II diabetes [78, 79]. Single-nucleotide polymorphisms of IL-1*β*, which may elevate circulating IL-1*β*, are frequently observed in patients with metabolic syndrome including atherosclerosis [85, 86] and NASH [87]. In addition to these findings, blockade of IL-1*β* decreased the severity of atherosclerosis and insulin sensitivity in animal models [88, 89]. HF diet feeding, a diet model for obesity and hepatic steatosis, results in severe steatohepatitis in IL-1 receptor antagonist-deficient mice [90], suggesting that IL-1*β* plays an important role in NASH. The proposed functions of IL-1*β* are as follows: (1) lipid accumulation in hepatocytes [26, 71], (2) hepatocyte cell death [26], and (3) activation of hepatic stellate cells [26, 57, 58]. IL-1*β* promotes hepatic steatosis by activating PPAR*α* [62] and diacylglycerol acyltransferase 2, an enzyme that converts diglyceride to triglyceride [26]. In addition, IL-1*β* promotes cell death in lipid-accumulated hepatocytes. Upon IL-1*β* stimulation, antiapoptotic genes are upregulated in normal hepatocytes. In contrast, proapoptotic genes such as Bax are induced in lipid-accumulated hepatocytes treated with IL-1*β* [26]. IL-1*β* induces the production of nitric oxide, generating peroxynitrite in the presence of superoxide radicals, which induces hepatocellular injury. In NAFLD, free radicals are generated by *β*-peroxidation of FFAs, and nitric oxide metabolites are increased in rats with NASH [91], which may further promote liver injury. Moreover, IL-1*β* contributes to liver fibrosis by activating hepatic stellate cells [26, 57, 58]. IL-1*β* induces the expression of TIMP-1 and TGF*β* in hepatic stellate cells. We have shown that IL-1R-deficient mice are resistant to CDAA diet-induced liver fibrosis [26]. Thus, IL-1*β* is an important factor in the development of NAFLD.

## 9. Chemokines and NAFLD

 Chemokines, strongly induced by TLR stimulation, play an important role in the development of metabolic syndrome including NAFLD. TLR4- and MyD88-deficient mice, which are resistant to metabolic syndrome, show reduced chemokine production compared with WT mice [39, 40]. MCP-1 levels are elevated in genetically obese diabetic (db/db) mice and in HF diet-fed mice. This suggests that MCP-1 and its receptor CCR2 contribute to the metabolic syndrome including obesity-related steatosis [92–95]. Indeed, MCP-1 overexpressing transgenic mice exhibit insulin resistance and hepatic steatosis as well as macrophage infiltration in adipose tissue. In contrast, MCP-1- or CCR2-deficient mice have attenuated HF diet-induced steatosis and macrophage infiltration. In these mice, inflammatory cytokine production is reduced, which could ameliorate steatohepatitis. Moreover, administration of a CCR2 antagonist improves insulin resistance. Clinical studies also demonstrate that MCP-1 levels in adipose tissue positively correlate with BMI, and patients with type II diabetes have higher serum MCP-1 levels than nondiabetes [96].

 In addition to macrophage recruitment, MCP-1 promotes hepatic lipid accumulation by increasing lipid synthesis and by inhibiting lipid efflux from hepatocytes [97]. MCP-1 increases PEPCK level, resulting in de novo lipogenesis. MCP-1 decreases secretion of ApoB, which suppresses lipid efflux. Since hepatocytes do not express CCR2, hepatocytes may utilize other receptors such as CCR7 and CCR8 as the receptors for MCP-1. Thus, MCP-1 regulates lipid metabolism through macrophage recruitment and also directly on hepatocytes.

## 10. Perspectives

 This paper summarized the role of TLRs and their downstream molecules in the development of NAFLD and showed that TLR signaling mediates steatosis, inflammation, and fibrosis. Thus, regulation of TLRs and their downstream molecules is potential targets for the therapy of NAFLD, in particular NASH. Several antagonists for TNF*α*, IL-1*β*, and CCR2 are used in NAFLD animal models [89, 93, 98–101]. In the future, these agents may be new tools for the therapy of human NAFLD. In addition to the blockade of TLR signaling, control of TLR ligands is another option for the therapy of NAFLD. Probiotics may suppress the growth of harmful intestinal bacteria and the generation of TLR ligands in the intestine. As a result, exposure to TLR ligands may be decreased in the liver. Beneficial effects of probiotics have been reported in animal NAFLD models [98, 102, 103]. Since their adverse effects are minimal in humans, randomized clinical trials of adequate size and methodology are needed for assessing the benefit of using probiotics on the NAFLD patients. 

 TLRs play multiple roles in multiple steps and in many hepatic cells in the development of NAFLD. In this review, we focused on the TLR signaling in Kupffer cells that produce key mediators in NAFLD. Other resident liver cells and recruited immune cells also produce many mediators that modulate the status of NAFLD in response to TLR ligands (Figure 1). Thus, better understanding of TLR signaling will provide new insight into the management and prevention of NAFLD.

## Abbreviations

CCR: C-C chemokine receptor
CDAA: Choline-deficient amino-acid defined
CSAA: Choline-supplemented amino acid defined
FFA: Free fatty acid
HF: High fat
HOMA-IR: Homeostasis model assessment of insulin resistance
IL: Interleukin
IL-1R: IL-1 receptor
JNK: c-Jun N-terminal kinase
LPS: Lipopolysaccharide
MCD: Methionine and choline deficient
MCP-1: Monocyte chemoattractant protein-1
NAFLD: Nonalcoholic fatty liver disease
NASH: Nonalcoholic steatohepatitis
NEMO: NF-*κ*B essential modulator
NF-*κ*B: Nuclear factor *κ*B
TNF: Tumor necrosis factor
TLR: Toll-like receptor
WT: Wild type.
